# Antibiotics in Chronic Liver Disease and Their Effects on Gut Microbiota

**DOI:** 10.3390/antibiotics12101475

**Published:** 2023-09-22

**Authors:** Nahum Mendez-Sanchez, Carlos Esteban Coronel-Castillo, Jacqueline Cordova-Gallardo, Xingshun Qi

**Affiliations:** 1 Unit Liver Research, Medica Sur Clinic & Foundation, Mexico City 14050, Mexico; 2 Faculty of Medicine, National Autonomous University of Mexico, Mexico City 04510, Mexico; 3 Internal Medicine Section, Central Military Hospital, Mexico City 11200, Mexico; 4 Department of Hepatology, Service of Surgery and Obesity Clinic, General Hospital “Dr. Manuel Gea González”, Mexico City 14080, Mexico; 5 Department of Gastroenterology, General Hospital of Northern Theater Command, Shenyang 110840, China

**Keywords:** dysbiosis, gut microbiota, liver cirrhosis, antibiotics, bile acids, PAMPs, metabolic dysfunction, inflammation

## Abstract

Impairments in liver function lead to different complications. As chronic liver disease progresses (CLD), hypoalbuminemia and alterations in bile acid compositions lead to changes in gut microbiota and, therefore, in the host–microbiome interaction, leading to a proinflammatory state. Alterations in gut microbiota composition and permeability, known as gut dysbiosis, have important implications in CLD; alterations in the gut–liver axis are a consequence of liver disease, but also a cause of CLD. Furthermore, gut dysbiosis plays an important role in the progression of liver cirrhosis and decompensation, particularly with complications such as hepatic encephalopathy and spontaneous bacterial peritonitis. In relation to this, antibiotics play an important role in treating CLD. While certain antibiotics have specific indications, others have been subjected to continued study to determine whether or not they have a modulatory effect on gut microbiota. In contrast, the rational use of antibiotics is important, not only because of their disrupting effects on gut microbiota, but also in the context of multidrug-resistant organisms. The aim of this review is to illustrate the role of gut microbiota alterations in CLD, the use and impact of antibiotics in liver cirrhosis, and their harmful and beneficial effects.

## 1. Introduction

The human microbiota refers to the living microorganisms that colonize our body. However, the microbiome refers not only to this collection of living microorganisms, but also to their genomes and products, such as structural elements and metabolites, and even environmental conditions. The microbiome colonizes our body from birth, and it undergoes a dynamic process of shaping and multiplication, with modifications in composition depending on genetic, nutritional, and environmental factors [1,2]. The composition of the human microbiome varies from site to site; it is highly diverse and comprises trillions of microorganisms. The gut microbiome has the highest number of microorganisms and has been extensively studied because of its impacts on health and disease [3]. Moreover, the interactions of the gut microbiome (GM) with different organs and systems each have a unique profile. Modifications in GM composition and function induce intestinal permeability, changes in digestion and metabolism, and immune responses. Misbalances in GM can lead to the onset and worsening of many diseases—not only gastrointestinal, but also metabolic, immunological, and neuropsychiatric [4,5,6].

In liver disease, interactions within the gut–liver axis are especially important, not only in relation to the decompensation and progression of liver cirrhosis, but, as stated before, in metabolic diseases. In this regard, there is growing evidence regarding the impacts of GM on metabolic syndrome (MetS) and the development of metabolic dysfunction-associated fatty liver disease (MAFLD) [3,4,6,7]. The gut–liver axis exhibits a reciprocal interaction facilitated by the portal vein, which enables the transport of gut-derived products directly to the liver, and the feedback of liver bile and antibody secretion to the intestine [7]. Therefore, a microbiome that is altered, or not, by disease will lead to portal dissemination of pathogen-associated molecular patterns (PAMPs), and other microbe-derived metabolites, such as trimethylamine and secondary bile acids (BAs). The liver may release inflammatory ligands, extracellular vesicles (EVs) that act as damage-associated molecular patterns (DAMPs), which change the BA composition and output. Given these interactions, many therapies that include probiotics, prebiotics, fecal microbial transplantation, and metabolic targets such as FXR agonists are currently being tested in different liver diseases [7,8,9]. Therefore, antibiotics have been used in chronic liver disease; the rationale for antibiotic use in these patients is to prevent the production and absorption of gut-derived neurotoxins (e.g., ammonia) and to reduce inflammation, keeping in mind that these antibiotics can have several negative effects on the gut microbiota, including reduced species diversity, altered metabolic activity, and the selection of antibiotic-resistant organisms [8,9]. This review will address the role of GM in liver disease and the role of antibiotics as therapeutics, but also as major disruptors of GM. 

## 2. Overview of Gut Microbiome Functions and Alterations in Liver Disease

GM composition includes bacteria, viruses, fungi, and parasites. It is different in each individual and there is not an optimal one. However, a healthy GM must have a balance, in order to optimally perform, between metabolic and immune functions. Despite GM variability, the dominant gut microbial phyla are Firmicutes, Bacteroidetes, Actinobacteria, Proteobacteria, Fusobacteria, and Verrucomicrobia. In adults, Firmicutes are the most abundant, followed by Bacteroidetes and Actinobacteria [1,10,11].

The main functions of GM are, but are not limited to, nutrient metabolism, xenobiotic and drug metabolism, immunomodulation, antimicrobial protection, and the metabolism of enzymes and other organic substances such as urea and BAs [12,13]. All those functions play an important role in maintaining body homeostasis. For instance, Bacteroides, Roseburia, Bifidobacterium, Fecalibacterium, and Enterobacteria are related to the production of SCFAs, such as acetate, propionate, and butyrate, that regulate lipogenesis and cholesterol biosynthesis in the liver [11], while Bacteroides are involved in synthesis of vitamin K [13]. Regarding the metabolism of BAs, Bacteroides also conjugate linoleic acid (CLA), and deconjugate and dehydrate the primary BAs and convert them into the secondary Bas, deoxycholic and lithocolic acids, in the colon [13,14].

Concerning immune functions, it is important to remember that the gut has the largest lymphoid tissue in the body. The mucus layer and the intestinal epithelium together constitute the physical barrier to gut microbes, whereas the immune cells of the lamina propria act as the immunological barrier. Regarding the latter, immune cells in the gut, including B and T lymphocytes, macrophages and antigen-presenting cells, alongside a collection of multi-follicular structures, including the tonsils, Peyer’s patches, appendix, colonic and cecal patches, and a number of smaller, isolated lymphoid follicles (ILF), conform to the gut-associated lymphoid tissues (GALT) [15,16,17]. Therefore, there is a constant and active interaction between gut microbiota and the immune system, which maintains eubiosis and immune homeostasis.

Furthermore, GM is important for the development of a capable immune system; studies in germ-free mice, which have no intestinal bacteria, have demonstrated a dramatic reduction in the size of GALT [18]. In addition, there is competition for nutrients, which stimulates innate immunity through the secretion of IgA and the activation of Toll-like receptors (TLRs) by compounds from active microorganisms, which, structurally, include lipopolysaccharide of bacterial origin (LPS), lipoproteins, flagellin, and DNA of pathogen-associated molecular patterns (PAMPs). These may act as disease triggers and mediators even in the absence of a previous condition when significant alterations in GM composition are caused by several factors. In fact, significant and constant changes in the diet or in the substances that are released in the gut will eventually alter GM composition and the way immune cells and microorganisms interact. For example, trimethylamine N-oxide (TMAO) is derived from the conversion of choline, mainly by Desulfovibrio desulfuricans and Escherichia coli. The increased synthesis of TMAO due to the overgrowth of these bacteria results in a lack of choline in the body, which, in turn, enhances oxidative stress in hepatocytes and increases liver inflammation and fibrosis [19,20]. 

In the context of chronic liver disease, patients have lower levels of Bacteroidetes and higher levels of Proteobacteria, Enterococcus, Veillonella, Megasphaera, Burkholderia, Prevotella and Fusobacteria. These changes in gut microbiota composition are, among other things, mainly related to alterations in BAs, lipid metabolism and the activation of inflammatory pathways. In fact, patients with liver fibrosis exhibit an altered BA profile that can change the composition of the gut microbiota and exacerbate fibrosis [21,22]. 

BAs have direct cytotoxicity and antibacterial activity, but also indirectly mediate the inhibition of microbial growth by regulating the expression of nitric oxide synthase and antimicrobial peptide genes. When BAs bind to FXR, antimicrobial peptides, such as angiogenin 1, are produced. These peptides can inhibit gut microbiota growth by increasing the intestinal epithelial cell potential to prevent bacterial uptake, improving gut barrier function. An increase in the harmful bacterial release of PAMPs into enterohepatic circulation in the context of a disrupted intestinal barrier induces the activation of immune cells in the liver. Chemokines, such as CC-chemokine ligand 2 (CCL2) and IL8, recruit immune cells, such as macrophages and neutrophils, to the liver. Another important cytokine is IL1β, which is induced by NF-kB following the activation of TLR4 by PAMPs such as LPS [23,24,25].

Furthermore, in patients with cirrhosis, this same mechanism can increase decompensation episodes, such as spontaneous SBP, and the risk of acute-on-chronic liver failure (ACLF) [25,26]. 

On the other hand, an altered, but not necessarily reversed, GM can mediate liver diseases. As stated before, the GM can influence the size and composition of the BA pool through the conversion of primary to secondary BAs, which act as signaling molecules affecting lipid and glucose metabolism, and predisposing individuals to metabolic diseases. In patients with MASLD, studies found that high levels of serum GCA and stool DCA are related to severe fibrosis and are positively correlated with Lachnospiraceae and negatively correlated with Bacteroidaceae levels. Another example of the impact of lipid metabolism is the reduction in SCFA-producing microbiota such as Bacteroidaceae, since SCFAs protect the intestinal barrier and prevent the development of MASLD by their effects on free fatty acid metabolism and visceral adipose tissues levels, reducing TNF expression and the activation of the NF-κB pathway [27,28]. 

Finally, patients with liver cirrhosis require different therapeutic options due to episodes of hepatic decompensation, which are particularly related to dysbiosis. This is the case of HE, wherein Rifaximin is the cornerstone treatment not only in acute events but also in preventing future episodes, since the overgrowth of ammonia-producing bacteria occurs in the gut. While this antibiotic seems to be more beneficial than harmful, there is no doubt that it has important effects on the GM [28,29]. 

## 3. Reevaluating the Therapeutic Use of Antibiotics in Liver Cirrhosis

**Figure 1:**
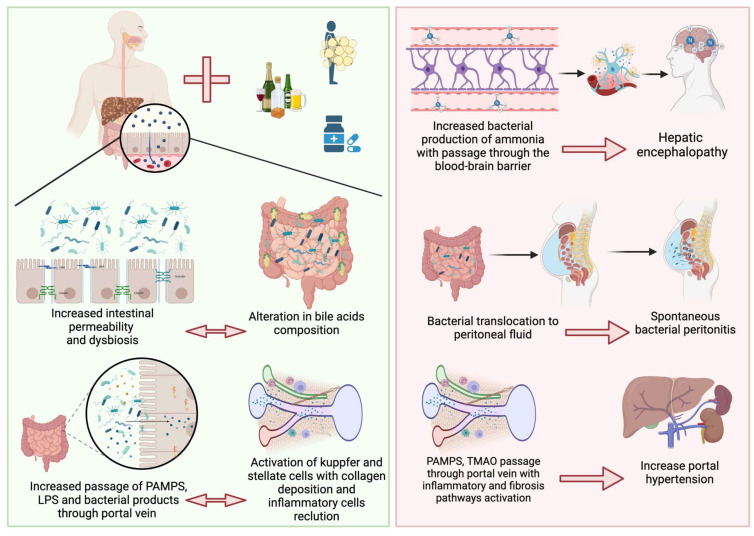
In chronic liver diseases, dysbiosis and increased bacterial overgrowth provoke altered bile acid composition associated with reduced intestinal motility and the expression of tight junction proteins produces leaky gut. This leads to the increased passage of PAMPs, LPS, and bacterial products to the liver through the portal vein, with the consequent activation of inflammatory pathways. All of these alterations lead to increased ammonia production with consequent hepatic encephalopathy; the translocation of bacteria into the peritoneal fluid leading to spontaneous bacterial peritonitis; and the activation of proinflammatory and fibrotic pathways with increased hepatic vascular tone leading to portal hypertension.

Patients with cirrhosis are predisposed to bacterial infections; an example is the high prevalence of Spontaneous Bacterial Peritonitis (SBP). In addition, these patients exhibit small intestinal bacterial overgrowth, increased intestinal permeability and reduced intestinal motility that may be related to the severity and progression of liver disease. Moreover, Prado et al. [29] conducted a study aimed at determining whether rectal colonization by resistant bacteria increased the likelihood of subsequent infection by the same strain in critically ill patients with cirrhosis. The presence of resistant bacteria in the rectal flora was assessed through rectal swab samples. The findings suggest that rectal colonization serves as a reservoir for potential infections, particularly when the colonizing bacteria are resistant to antibiotics. This phenomenon is of particular concern in critically ill patients with cirrhosis, as their compromised immune system and impaired liver function contribute to increased vulnerability to infections [29,30] (Figure 1).

Furthermore, SBP, urinary tract infections, and pneumonia are the most common infections in patients with liver cirrhosis. Current evidence implies that about 48% of infections in liver cirrhosis are community-acquired, while 52% are related to nosocomial factors, and are healthcare-related. In this matter, there is great concern regarding multidrug-resistant organisms (MDROs) [31,32,33]. Since antibiotics are frequently prescribed, a group of researchers conducted the ATTIRE trial to assess the impact of antibiotic therapy in patients with decompensated cirrhosis. Through a randomized controlled trial design, they compared the outcomes of patients who received early antibiotic treatment upon hospital admission to those who received antibiotics only if an infection was clinically suspected. The results of the study revealed no significant difference in overall survival between the two groups, challenging the routine use of early antibiotic therapy in this patient population [33,34]. Furthermore, Bajaj and colleagues found that prophylactic antibiotics may disrupt the natural phage–bacterial balance, leading to shifts in phage populations and potentially affecting microbial diversity [34].

Another common scenario of the interplay between dysbiosis and chronic use of antibiotics in CLD is HE. Historically, metronidazole, neomycin, and vancomycin have been used to treat HE, but these are currently no longer used due to their side effects and the growing prevalence of MDROs [35]. In contrast, rifaximin is the preferred option for HE due to its proven safety and efficacy. Nevertheless, this drug is not exempt from the MDRO issue, exemplified by E. coli-resistant strains [36,37]. Furthermore, while rifaximin is a classical positive modulator of GM, acting by maintaining gut microbiota diversity and composition and not changing the overall resistome, this continues to be questioned [38,39].

While antibiotic prophylaxis has proven beneficial, the careful consideration of individual patient characteristics is essential. Factors such as antibiotic resistance patterns, renal function, and the presence of comorbidities should be evaluated when selecting the appropriate prophylactic regimen. To further address the issue of the importance of antibiotics in the context of liver disease in contrast to their harmful effects, we summarize their clinical use below. 

### 3.1. Antibiotic Effects on Portal Hypertension

The portal vein serves as a major conduit for nutrients, toxins, and microbial products from the gut to the liver. Disruption of the gut–liver axis can lead to dysbiosis, which has been implicated in the pathogenesis of CLD. Studies have shown that alterations in gut microbiota composition and function contribute to liver inflammation, fibrosis, and portal hypertension. The dysbiosis-induced increased intestinal permeability to gut microbial metabolites, such as LPS, secondary BAs, and TMAO, has been shown to influence hepatic vascular tone and contribute to portal hypertension [40]. Moreover, evidence suggests that when those metabolites escape to the systemic circulation, they may induce systemic hypertension [41,42]. 

Recent research on factors influencing GM with regard to portal hypertension has opened new avenues for therapeutic interventions. Modulating the gut microbiota through strategies such as probiotics, prebiotics, antibiotics, and fecal microbiota transplantation might represent promising therapies to improve liver-related complications and reduce portal hypertension. Additionally, targeting gut microbial metabolites and their receptors may offer novel therapeutic options for the management of portal hypertension [40,43]. In fact, bacterial-derived products may increase hyperdynamic circulation and intrahepatic vascular resistance, promoting a further increase in portal pressure and the risk of bleeding [44,45,46].

Regarding infections, when compared with controls, patients with liver cirrhosis and increased populations of Bacteroides, Escherichia, Shigella, and Prevotella have severe portal hypertension and high levels of IL-8 in their hepatic veins [47]. Furthermore, it seems that patients with variceal bleeding have a higher rate of bacterial infections, and the administration of intravenous antibiotics, such as norfloxacin or ampicillin/sulbactam, may improve complications [45].

**Figure 2:**
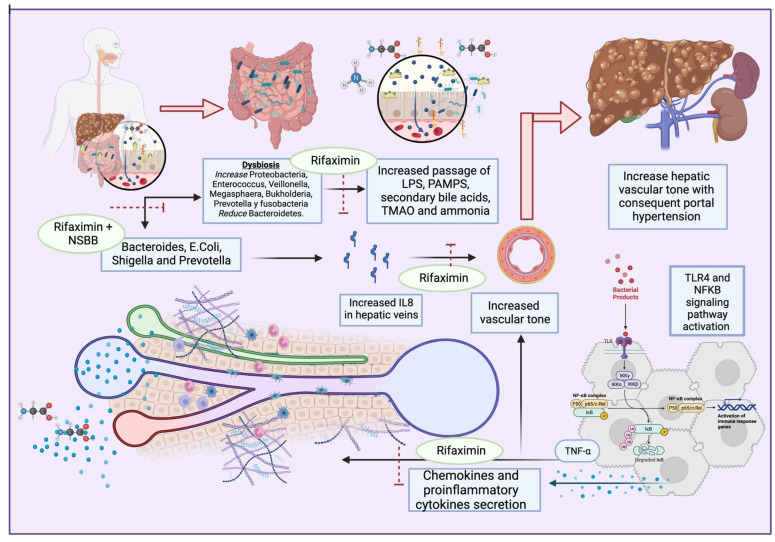
Dysbiosis enhances the secretion of PAMPs, secondary bile acids, TMAO, and ammonia, and activates TLR4 and NFkB pathways. This results in proinflammatory cytokine and chemokyne secretion, with increases in TNFα and IL8 that lead to portal hypertension. Antibiotics such as rifaximin seem to exert beneficial effects on multiple dysbiosis-reducing targets, IL8-producing bacteria, and the passage of bacterial products, with consequent proinflammatory pathway activation.

A recent study published by Mendoza et al [48]. showed that the use of rifaximin or norfloxacin did not cause a significant reduction in hepatic venous pressure gradient (HPVG) in patients with cirrhosis, but the use of antibiotics for longer periods in association with non-selective beta blockers (NSBB) did decrease HPVG significantly [48]. The use of rifaximin has been shown to reduce portal hypertension when associated with NSBB, compared to the use of propranolol alone [49] (Figure 2). However, norfloxacin did not perform better than the placebo in reducing HVPG [50]. Moreover, the use of probiotic VSL#3 has been shown to improve the effect of propranolol in reducing HPVG [51].

### 3.2. Prophylactic Antibiotic Use for Cirrhosis

Current guidelines recommend antibiotic prophylaxis in specific situations. For patients with a history of SBP, long-term prophylaxis with oral norfloxacin or trimethoprim–sulfamethoxazole is recommended to prevent recurrence. Additionally, short-term prophylaxis with intravenous antibiotics is advised for cirrhotic patients with gastrointestinal bleeding, as it reduces the risk of infections and improves survival rates [52,53]. Regarding the latter, consensus guidelines recommend the prophylactic use of oral or intravenous antibiotics in this population. Furthermore, quinolones and beta-lactams, either alone or in combination, were effective in reducing rebleeding rates and hospital stay length in cirrhosis patients with gastrointestinal bleeding, according to a metanalysis. On the other hand, MDRO bacterial infections have reduced the efficacy of commonly used antibiotics, necessitating combined antibiotic therapy. Combination therapy with quinolones and beta-lactams has been associated with reduced mortality, rebleeding, and hospitalization lengths [53].

Patients with liver cirrhosis experience about 36% spontaneous infections, such as with SBP [54]. When SBP is suspected, empiric antibiotics are used, with third-generation cephalosporins used commonly, except in the context of MDRO risk factors, where the first option is piperacillin/tazobactam. In the case of prophylaxis, norfloxacin and ciprofloxacin are the first options for both primary and secondary prevention, followed by trimethoprim–sulfamethoxazole [55,56,57,58]. The empirical antibiotics discussed above seem to exert similar effects against SBP, but response-guided therapy, by performing a second paracentesis at 48 h to assess antibiotic response, should be considered [56]. The use of prophylactic norfloxacin might increase the risk of MDR bacterial infections, and practitioners should be aware of this after the first month of liver transplantation [59]. Hence, MDR bacterial infection remains controversial, so norfloxacin prophylaxis should be indicated in carefully selected patients [60]. 

Another novel strategy is selective digestive decontamination (SDD), which consists of the combination of topical nonabsorbable antibiotics or antifungal agents applied to the upper gastrointestinal tract with a short course of intravenous antibiotics. Its use began in patients with neutropenia, and it is a topic of interest in critically ill patients despite controversial evidence [61,62]. In cirrhosis, SDD was used to treat both gastrointestinal bleeding and SBP, at first with oral nonabsorbable antibiotics such as polymyxin, neomycin, gentamycin and colistin, and then with trimethoprim–sulfamethoxazole and fluroquinolones. Still, the disrupting effects of antibiotics in GM may be linked to the asymptomatic colonization of the gut by MDROs. This colonization not only represents a potential source of infection for the affected patient, but also contributes to the transmission of MDRO infections within healthcare settings. Consequently, until comprehensive studies have been conducted across multiple centers, investigating the impact of SDD on rates of multidrug resistance at both the individual and population levels, the use of SDD should be restricted to cirrhosis patients who face the highest risk of developing an infection [63]. To address this issue, the use of rifaximin is proposed; this non-absorbable antibiotic possesses distinctive effects on the gut microbiota [58]. However, the results of a recent study found that, overall, systemic antibiotic prophylaxis is more effective than rifaximin in SBP prevention and should be the standard of care for patients with advanced cirrhosis and a high risk of SBP [64].

Finally, rifaximin, in combination with lactulose or L-ornithine L-aspartate, is employed for the purpose of preventing the recurrence of HE [65,66]. According to research findings, it seems that rifaximin enhances the population of beneficial intestinal bacteria, such as Bifidobacterium, Atopobium, and Faecalibacterium prausnitzii. Meanwhile, it does not significantly alter the overall composition of the gut microbiota, including the lactobacilli. Additionally, rifaximin contributes to the restoration of the intestinal barrier, potentially mitigating bacterial translocation and systemic endotoxemia in individuals with cirrhosis. This effect may be attributed to the inhibition of NF-kB activation via the pregnane X receptor (PXR) and a reduction in interleukins and TNFα expression [39,59,67] (Table 1).

### 3.3. Multidrug-Resistant Bacterial Infections in Patients with Cirrhosis and the Role of Gut Microbiota

Bacterial infections represent one of the leading causes of hospitalization, morbidity, and mortality in cirrhotic patients. The most frequent infections are urinary infections, pneumonia, and spontaneous bacterial peritonitis, with an increasing incidence of MDROs [31].

Owing to the increasing use of broad antibiotics in cirrhotic patients, multidrug-resistant bacterial infections have been rising; in particular, patients who received prophylactic norfloxacin for SBP experience higher risks of MDRO infection [68]. Hence, this assertion remains controversial; in a study performed by Marciano et al., they found that norfloxacin exerts a beneficial effect on SBP prophylaxis, with no increased incidence of MDRO infections [60]. To address the uncertainty as to whether antibiotic prophylaxis is beneficial or not, more clinical trials should be performed to test long-term antibiotics [69]. Furthermore, in a multicenter study in Europe, it was found that about 30% of positive cultures from infections in patients with liver cirrhosis were caused by MDROs. The most frequently isolated MDROs in this series were extended-spectrum beta-lactamase-producing Enterobacteriaceae. In that same study, in a second series of patients it was revealed that the prevalence of MDROs was 23% (392 infections out of 2587 patients), and among culture-positive infections, it was 38%. A slight increase in the rate of carbapenem-resistant Enterobacteriaceae was observed in this series [77]. In general, a global prevalence of 34% MDR bacterial infection is estimated in liver cirrhosis [32]. Antibiotic resistance is associated with poor prognosis and the failure of antibiotic strategies, particularly those based on third-generation cephalosporins or quinolones [78]. Furthermore, the main risk factors for MDRO infections in patients with cirrhosis are long-term norfloxacin prophylaxis, recent infection by multi-resistant bacteria, and the recent use of β-lactams [79]. 

It is important to consider the spectrum of infectious pathogens from Gram-negative bacteria in community-acquired infections compared with Gram-positive bacteria in hospital-acquired infections [80].

Antibiotics may also predispose individuals to other infections, such as invasive fungal infections. Fungal infections are much less frequent; they are usually nosocomial and associated with extremely high short-term mortality. In patients with cirrhosis, invasive fungal infections occur in approximately 3–7% of culture-positive infected individuals, and they are more commonly observed as secondary or nosocomial infections during the course of acute-on-chronic liver failure (ACLF). Among them, invasive candidiasis, or candidemia, is the most frequent, accounting for 70–90% of cases, followed by invasive aspergillosis. 

Invasive fungal infections in patients with decompensated cirrhosis are generally associated with an extremely poor prognosis. Candidemia and other invasive candidiasis infections are accompanied by 28-day mortality rates ranging from 45% to 60%. ACLF complicated by IA has an even worse prognosis, with only rare cases of survival despite receiving appropriate antifungal treatment [81].

Using a targeted metagenomics approach, Delavy et al. [82] observed a high degree of interindividual diversity in healthy gut microbiota. They found that the prevalence of *C. albicans* was much higher than previously reported, with all subjects except one carrying *C. albicans*, albeit at varying levels. The administration of third-generation cephalosporins significantly altered the composition of the microbiota, and the fungal load was increased both in the short and the long term. The variations in *C. albicans* levels in response to third-generation cephalosporin treatment could be partially explained by changes in the levels of endogenous fecal β-lactamase activity. Subjects with higher β-lactamase activity showed lower *C. albicans* levels [82]. This suggests that the use of a particular antibiotic treatment may change the specific types of microorganisms, either fungal or bacterial, in the GM [83].

## 4. Conclusions

The use of antibiotics, mainly rifaximin, can be beneficial in reducing inflammation and liver fibrosis, thus modifying the gut microbiota, and could exert a reducing effect on portal hypertension when associated with NSBB. The use of norfloxacin for the primary or secondary prophylaxis of SBP is controversial and should be enforced on a case-by-case basis, but it could have favorable effects on survival and SBP incidence and recurrence rates. Nonetheless, the excessive growth of MDROs should be considered by physicians to inform rational use. Rifaximin has shown several beneficial effects, including reducing HPVG when associated with NSBB, reducing ammonia-producing bacteria (thus improving hepatic encephalopathy), and reducing intestinal permeability and dysbiosis; therefore, reducing the passage of PAMPs decreases liver inflammation and probably liver fibrosis, in turn reducing SBP incidence. For these reasons, the use of antibiotics in patients with cirrhosis should aim to reduce the incidence of MDROs. 

## Figures and Tables

**Table 1:** Antibiotics used in chronic liver disease clinical trials and meta-analysis results.

Study	Type of Study	Drug	Number of Patients	Outcomes	Conclusion
Effect of Prophylactic Antibiotics on Mortality in Severe Alcohol-Related Hepatitis: A Randomized Clinical Trial [68]	multicenter, randomized, double-blind clinical trial	amoxicillin-clavulanate, compared with placebo	145 amoxicillin-clavulanate, 147 placebo	no significant difference in 60-, 90- or 180-day mortality, infection rate lower in amoxicillin-clavulanate group	amoxicillin-clavulanate combined with prednisolone did not improve survival compared with prednisolone alone
Impact of Prophylactic Norfloxacin in Multidrug Resistant Bacterial Infections in the Early Liver Posttransplant Period [59]	prospective cohort study	norfloxacin	157 liver recipients: 54 received norfloxacin and 103 did not	incidence of multidrug-resistant bacterial infection was higher in the norfloxacin group	higher risk of MDROs infections during the first month after liver trasplant in patients who received prophylactic norfloxacin
Response-Guided Therapy With Cefotaxime, Ceftriaxone, or Ciprofloxacin for Spontaneous Bacterial Peritonitis: A Randomized Trial: A Validation Study of 2021 AASLD Practice Guidance for SBP [56]	multicenter, prospective, randomized–controlled trial	cefotaxime, ceftriaxone and ciprofloxacin	261 patients	resolution rates at 120 h were similar among the groups, as was the 1-month mortality	the efficacy of empirical antibiotics was similar, based on response-guided therapy, and should be insured
Chronic Rifaximin Use in Cirrhotic Patients Is Associated with Decreased Rate of C. difficile Infection (CDI) [58]	retrospective	rifaximin	701 patients	rifaximin use in cirrhotic patients reduced CDI infection	patients with cirrhosis that were chronically receiving rifaximin have lower rates of CDI
Norfloxacin Prophylaxis Effect on Multidrug Resistance in Patients with Cirrhosis and Bacterial Infections [69]	cross-sectional study	norfloxacin	472 patients	13 (24.5%) patients with norfloxacin and 90 (21.5%) of those not receiving it presented MDROs infections	norfloxacin prophylactic use was not associated with multidrug-resistant bacterial infections
Evaluating the Role of Antibiotics in Patients Admitted to Hospital With Decompensated Cirrhosis: Lessons From the ATTIRE Trial [34]	clinical trial (ATTIRE patients without infection at baseline grouped by antibiotic prescription or not)	antibiotics use vs. non-antibiotics	408 patients	long-term antibiotic prophylaxis at discharge showed no differences in 6-month mortality	prompt antibiotic de-escalation or discontinuation is recommended guided by culture sensitivities at 24–48 h after commencement if no infection is confirmed
Meta-analysis: Antibiotic Prophylaxis for Cirrhotic Patients with Upper Gastrointestinal Bleeding—an Updated Cochrane Review [45]	meta-analysis of randomized clinical trials	antibiotic vs. no antibiotic prophylaxis	1241 patients	antibiotic prophylaxis was associated with beneficial effects on mortality, bacterial infections, rebleeding and hospitalization length, with no adverse events	in cirrhotic patients with upper gastrointestinal bleeding, prophylactic antibiotic use significantly reduced bacterial infections, all-cause mortality, events, and hospitalization length
Antibiotic Prophylaxis for Upper Gastrointestinal Bleed in Liver Cirrhosis; Less May Be More [70]	retrospective cohort study	antibiotic prophylaxis for upper gastrointestinal bleeding	243 patients (77 received antibiotics for <3 days, 69 patients 4–6 days, and 97 >6 days)	rates of infection were not statistically different among the groups; 11 patients developed pneumonia, 8 developed UTI, 4 developed SBP, and 3 developed bacteremia within the 30 days following GI bleeding	if there is no active infection, a short course of prophylactic antibiotics (3 days) is preferred in patients with upper GI bleeding
Efficacy of Norfloxacin Prophylaxis to Prevent Spontaneous Bacterial Peritonitis: A Systematic Review and Meta-Analysis [71]	meta-analysis of randomized controlled clinical trials	antibiotic prophylaxis for SBP	1626 patients	norfloxacin capacity to prevent SBP, but not death, was superior to placebo but decreased over time, and was not superior to other antibiotics	norfloxacin remained superior to placebo in preventing SBP
Efficacy and Safety of Alternating Norfloxacin and Rifaximin as Primary Prophylaxis for Spontaneous Bacterial Peritonitis in Cirrhotic Ascites: a Prospective Randomized Open-Label Comparative Multicenter Study [72]	randomized open-label comparative multicenter study	norfloxacin + rifaximin vs. norfloxacin or rifaximin alone	334 patients	alternating norfloxacin and rifaximin was the superior prophylactic treatment in reducing the probability of SBP	alternating the primary prophylaxis for SBP showed higher efficacy comparedwith monotherapy of norfloxacin
Randomized-Controlled Trial of Rifaximin ersus Norfloxacin for Secondary Prophylaxis of Spontaneous Bacterial Peritonitis [73]	randomized–controlled clinical trial	rifaximin vs. norfloxacin	262 patients	recurrence of SBP was significantly lower in the rifaximin group as well as mortality rate	rifaximin was more effective than norfloxacin in the secondary prevention of SBP
The Role of Rifaximin in the Primary Prophylaxis of Spontaneous Bacterial Peritonitis in Patients with Liver Cirrhosis [74]	retrospective clinical trial	rifaximin vs. non rifaximin	404 patients	reduction in SBP rate in rifaximin-treated patients	rifaximin may prevent SBP infections
Addition of Probiotics to Norfloxacin Does Not Improve Efficacy in the Prevention of Spontaneous Bacterial Peritonitis: a Double-Blind Placebo-Controlled Randomized-Controlled Trial [75]	double-blind placebo-controlled randomized–controlled trial	norfloxacin+ probiotics vs. norfloxacin + placebo	110 patients	rate of SBP, treatment failures, cumulative probability of mortality and side effects were similar among the groups	probiotics addition to norfloxacin prophylactic (primary or secondary) treatment did not reduce SBP frequency or mortality
Primary Prophylaxis of Spontaneous Bacterial Peritonitis Delays Hepatorenal Syndrome and Improves Survival in Cirrhosis [76]	randomized–controlled trial	norfloxacin	35 norfloxacin vs. 33 placebo	norfloxacin prophylactic treatment reduced 1-year probability of SBP and hepatorenal syndrome, as well as survival at 3 and 12 months.	norfloxacin primary prophylaxis reduces SBP and HRS incidence, as well as survival.
Effects of the Adjunctive Probiotic VSL#3 on Portal Hemodynamics in Patients with Cirrhosis and Large Varices: a Randomized Trial [51]	randomized double-blind placebo-controlled trial	probiotics VSL#3, norfloxacin	94 patients (3 groups: propranolol+placebo, propranolol+norfloxacin, propranolol+VSL#3)	adding probiotics and antibiotics to propranolol treatment reduces the mean HVPG and TNF alpha levels	adding VSL#3 probiotics improved propranolol therapy response rate
Norfloxacin Treatment for Clinically Significant Portal Hypertension: Results of a Randomized Double-Blind Placebo-Controlled Crossover Trial [51]	randomized double-blind placebo-controlled crossover trial	norfloxacin	16 patients	norfloxacin therapy was not superior to placebo in reducing HVPG	norfloxacin therapy was not superior to placebo in reducing HVPG but seems to modulate l-arginine transporter function
Rifaximin and Propranolol Combination Therapy Is More Effective than Propranolol Monotherapy for the Reduction of Portal Pressure: An Open Randomized Controlled Pilot Study [49]	randomized–controlledtrial	rifaximin	64 patients (propranolol vs. rifaximin vs. propranolol + rifaximin)	propranolol plus rifaximin was associated with better reduction in HVPG compared to propranolol alone	rifaximin in combination with propranolol had an additive effect in reducing portal hypertension

## References

[1] Rinninella E., Raoul P., Cintoni M., Franceschi F., Miggiano G.A., Gasbarrini A., Mele M.C. (2019). What is the Healthy Gut Microbiota Composition? A Changing Ecosystem across Age, Environment, Diet, and Diseases. Microorganisms.

[2] Peroni D.G., Nuzzi G., Trambusti I., Di Cicco M.E., Comberiati P. (2020). Microbiome Composition and Its Impact on the Development of Allergic Diseases. Front. Immunol..

[3] de Vos W.M., Tilg H., Van Hul M., Cani P.D. (2022). Gut microbiome and health: Mechanistic insights. Gut.

[4] Huo K., Wu Z.X., Chen X.Y., Wang J.Q., Zhang D., Xiao C., Zhu D., Koya J.B., Wei L., Li J. (2022). Microbiota in health and diseases. Signal. Transduct. Target. Ther..

[5] Fan Y., Pedersen O. (2021). Gut microbiota in human metabolic health and disease. Nat. Rev. Microbiol..

[6] Tang W.H., Kitai T., Hazen S.L. (2017). Gut Microbiota in Cardiovascular Health and Disease. Circ. Res..

[7] Albillos A., de Gottardi A., Rescigno M. (2020). The gut-liver axis in liver disease: Pathophysiological basis for therapy. J. Hepatol..

[8] Wang R., Tang R., Li B., Ma X., Schnabl B., Tilg H. (2021). Gut microbiome, liver immunology, and liver diseases. Cell. Mol. Immunol..

[9] Zoratti C., Moretti R., Rebuzzi L., Albergati I.V., Di Somma A., Decorti G., Di Bella S., Crocè L.S., Giuffrè M. (2022). Antibiotics and Liver Cirrhosis: What the Physicians Need to Know. Antibiotics.

[10] Beam A., Clinger E., Hao L. (2021). Effect of Diet and Dietary Components on the Composition of the Gut Microbiota. Nutrients.

[11] Olvera-Rosales L.B., Cruz-Guerrero A.E., Quintero-Lira A., Contreras-López E., Jaimez-Ordaz J., Castañeda-Ovando A., Añorve-Morga J., Calderón-Ramos Z.G., Arias-Rico J., González-Olivares L.G. (2021). Impact of the Gut Microbiota Balance on the Health–Disease Relationship: The Importance of Consuming Probiotics and Prebiotics. Foods.

[12] Valdes A.M., Walter J., Segal E., Spector T.D. (2018). Role of the gut microbiota in nutrition and health. BMJ.

[13] Jandhyala S.M., Talukdar R., Subramanyam C., Vuyyuru H., Sasikala M., Reddy D.N. (2015). Role of the normal gut microbiota. World J. Gastroenterol..

[14] Collins S.L., Stine J.G., Bisanz J.E., Okafor C.D., Patterson A.D. (2023). Bile acids and the gut microbiota: Metabolic interactions and impacts on disease. Nat. Rev. Microbiol..

[15] Okumura R., Takeda K. (2016). Maintenance of gut homeostasis by the mucosal immune system. Proc. Jpn. Acad. Ser. B Phys. Biol. Sci..

[16] Wong-Chew R.M., De Castro J.A., Morelli L., Perez M., Ozen M. (2022). Gut immune homeostasis: The immunomodulatory role of Bacillus clausii, from basic to clinical evidence. Expert. Rev. Clin. Immunol..

[17] Donaldson D.S., Else K.J., Mabbott N.A. (2015). The Gut-Associated Lymphoid Tissues in the Small Intestine, Not the Large Intestine, Play a Major Role in Oral Prion Disease Pathogenesis. J. Virol..

[18] Pollard M., Sharon N. (1970). Responses of the Peyer’s Patches in Germ-Free Mice to Antigenic Stimulation. Infect. Immun..

[19] Ruuskanen M.O., Åberg F., Männistö V., Havulinna A.S., Méric G., Liu Y., Loomba R., Vázquez-Baeza Y., Tripathi A., Valsta L.M. (2021). Links between gut microbiome composition and fatty liver disease in a large population sample. Gut Microbes.

[20] Cong J., Zhou P., Zhang R. (2022). Intestinal Microbiota-Derived Short Chain Fatty Acids in Host Health and Disease. Nutrients.

[21] Zheng Z., Wang B. (2021). The Gut-Liver Axis in Health and Disease: The Role of Gut Microbiota-Derived Signals in Liver Injury and Regeneration. Front. Immunol..

[22] Schwenger K.J., Clermont-Dejean N., Allard J.P. (2019). The role of the gut microbiome in chronic liver disease: The clinical evidence revised. JHEP Rep..

[23] Shao J.W., Ge T.T., Chen S.Z., Wang G., Yang Q., Huang C.H., Xu L.C., Chen Z. (2021). Role of bile acids in liver diseases mediated by the gut microbiome. World J. Gastroenterol..

[24] Yan S., Yin X.M. (2021). Gut microbiome in liver pathophysiology and cholestatic liver disease. Liver Res..

[25] Solé C., Guilly S., Da Silva K., Llopis M., Le-Chatelier E., Huelin P., Carol M., Moreira R., Fabrellas N., De Prada G. (2021). Alterations in Gut Microbiome in Cirrhosis as Assessed by Quantitative Metagenomics: Relationship With Acute-on-Chronic Liver Failure and Prognosis. Gastroenterology.

[26] Trebicka J., Macnaughtan J., Schnabl B., Shawcross D.L., Bajaj J.S. (2021). The microbiota in cirrhosis and its role in hepatic decompensation. J. Hepatol..

[27] Zhang Y.L., Li Z.J., Gou H.Z., Song X.J., Zhang L. (2022). The gut microbiota-bile acid axis: A potential therapeutic target for liver fibrosis. Front. Cell. Infect. Microbiol..

[28] Reuter B., Bajaj J.S. (2020). Microbiome: Emerging Concepts in Patients with Chronic Liver Disease. Clin. Liver Dis..

[29] Prado V., Hernández-Tejero M., Mücke M.M., Marco F., Gu W., Amoros A., Toapanta D., Reverter E., Peña-Ramirez C., Altenpeter L. (2022). Rectal colonization by resistant bacteria increases the risk of infection by the colonizing strain in critically ill patients with cirrhosis. J. Hepatol..

[30] Pande C., Kumar A., Sarin S.K. (2009). Small-intestinal bacterial overgrowth in cirrhosis is related to the severity of liver disease. Aliment. Pharmacol. Ther..

[31] Dalbeni A., Mantovani A., Zoncapè M., Cattazzo F., Bevilacqua M., De Marco L., Paon V., Ieluzzi D., Azzini A.M., Carrara E. (2023). The multi-drug resistant organisms infections decrease during the antimicrobial stewardship era in cirrhotic patients: An Italian cohort study. PLoS ONE.

[32] Piano S., Singh V., Caraceni P., Maiwall R., Alessandria C., Fernandez J., Soares E.C., Kim D.J., Kim S.E., Mariano M. (2019). Epidemiology and effects of bacterial infections in patients with cirrhosis worldwide. Gastroenterology.

[33] Bajaj J.S., Rodriguez M.P., Fagan A., McGeorge S., Sterling R.K., Lee H., Luketic V., Fuchs M., Davis B.C., Sikaroodi M. (2022). Impact of bacterial infections and spontaneous bacterial peritonitis prophylaxis on phage-bacterial dynamics in cirrhosis. Hepatology.

[34] Kutmutia R., Tittanegro T., China L., Forrest E., Kallis Y., Ryder S.D., Wright G., Freemantle N., O’Brien A. (2023). Evaluating the Role of Antibiotics in Patients Admitted to Hospital with Decompensated Cirrhosis: Lessons from the ATTIRE Trial. Am. J. Gastroenterol..

[35] Patidar K.R., Bajaj J.S. (2013). Antibiotics for the Treatment of Hepatic Encephalopathy. Metab. Brain Dis..

[36] Betts J.W., Phee L.M., Wareham D.W. (2016). Rifaximin combined with polymyxins: A potential regimen for selective decontamination of multidrug-resistant bacteria in the digestive tract?. J. Glob. Antimicrob. Resist..

[37] Kothary V., Scherl E.J., Bosworth B., Jiang Z.D., DuPont H.L., Harel J., Simpson K.W., Dogan B. (2013). Rifaximin Resistance in Escherichia coli Associated with Inflammatory Bowel Disease Correlates with Prior Rifaximin Use, Mutations in rpoB, and Activity of Phe-Arg-β-Naphthylamide-Inhibitable Efflux Pumps. Antimicrob. Agents Chemother..

[38] Bajaj J.S., Sikaroodi M., Shamsaddini A., Henseler Z., Santiago-Rodriguez T., Acharya C., Fagan A., Hylemon P.B., Fuchs M., Gavis E. (2021). Interaction of bacterial metagenome and virome in patients with cirrhosis and hepatic encephalopathy. Gut.

[39] Yu X., Jin Y., Zhou W., Xiao T., Wu Z., Su J., Gao H., Shen P., Zheng B., Luo Q. (2021). Rifaximin Modulates the Gut Microbiota to Prevent Hepatic Encephalopathy in Liver Cirrhosis Without Impacting the Resistome. Front. Cell. Infect. Microbiol..

[40] Arab J.P., Martin-Mateos R.M., Shah V.H. (2018). Gut-liver axis, cirrhosis and portal hypertension: The chicken and the egg. Hepatol. Int..

[41] Di Tommaso N., Santopaolo F., Gasbarrini A., Ponziani F.R. (2023). The Gut-Vascular Barrier as a New Protagonist in Intestinal and Extraintestinal Diseases. Int. J. Mol. Sci..

[42] Touyz R.M. (2021). Gut Dysbiosis–Induced Hypertension Is Ameliorated by Intermittent Fasting. Circ. Res..

[43] Li M., Li K., Tang S., Lv Y., Wang Q., Wang Z., Luo B., Niu J., Zhu Y., Guo W. (2022). Restoration of the gut microbiota is associated with a decreased risk of hepatic encephalopathy after TIPS. JHEP Rep..

[44] Lata J., Juránková J., Husová L., Senkyrík M., Díte P., Dastych M., Príbramská V., Kroupa R. (2005). Variceal bleeding in portal hypertension: Bacterial infection and comparison of efficacy of intravenous and per-oral application of antibiotics–a randomized trial. Eur. J. Gastroenterol. Hepatol..

[45] Chavez-Tapia N.C., Barrientos-Gutierrez T., Tellez-Avila F., Soares-Weiser K., Mendez-Sanchez, Gluud C., Uribe M.N. (2011). Meta-analysis: Antibiotic prophylaxis for cirrhotic patients with upper gastrointestinal bleeding–An updated Cochrane review. Aliment. Pharmacol. Ther..

[46] Zhang H., Gao J. (2022). Antibiotics and probiotics on hepatic venous pressure gradient in cirrhosis: A systematic review and a meta-analysis. PLoS ONE.

[47] Gedgaudas R., Bajaj J.S., Skieceviciene J., Varkalaite G., Jurkeviciute G., Gelman S., Valantiene I., Zykus R., Pranculis A., Bang C. (2022). Circulating microbiome in patients with portal hypertension. Gut Microbes.

[48] Mendoza Y.P., Rodrigues S.G., Bosch J., Berzigotti A. (2020). Effect of poorly absorbable antibiotics on hepatic venous pressure gradient in cirrhosis: A systematic review and meta-analysis. Dig. Liver Dis..

[49] Lim Y.L., Kim M.Y., Jang Y.O., Baik S.K., Kwon S.O. (2017). Rifaximin and Propranolol Combination Therapy Is More Effective than Propranolol Monotherapy for the Reduction of Portal Pressure: An Open Randomized Controlled Pilot Study. Gut Liver..

[50] Kemp W., Colman J., Thompson K., Madan A., Vincent M., Chin-Dusting J., Kompa A., Krum H., Roberts S. (2009). Norfloxacin treatment for clinically significant portal hypertension: Results of a randomised double-blind placebo-controlled crossover trial. Liver Int..

[51] Gupta N., Kumar A., Sharma P., Garg V., Sharma B.C., Sarin S.K. (2013). Effects of the adjunctive probiotic VSL#3 on portal haemodynamics in patients with cirrhosis and large varices: A randomized trial. Liver Int..

[52] Ferrarese A., Passigato N., Cusumano C., Gemini S., Tonon A., Dajti E., Marasco G., Ravaioli F., Colecchia A. (2021). Antibiotic prophylaxis in patients with cirrhosis: Current evidence for clinical practice. World J. Hepatol..

[53] Gao Y., Qian B., Zhang X., Liu H., Han T. (2022). Prophylactic antibiotics on patients with cirrhosis and upper gastrointestinal bleeding: A meta-analysis. PLoS ONE.

[54] Tay P.W.L., Xiao J., Tan D.J.H., Ng C., Lye Y.N., Lim W.H., Teo V.X.Y., Heng R.R.Y., Heng R.R.Y., Lum L.H.W. (2021). An Epidemiological Meta-Analysis on the Worldwide Prevalence, Resistance, and Outcomes of Spontaneous Bacterial Peritonitis in Cirrhosis. Front. Med..

[55] Biggins S.W., Angeli P., Garcia-Tsao G., Gines P., Ling S.C., Nadim M.K., Wong F., Kim W.R. (2021). Diagnosis, Evaluation and Management of Ascites, Spontaneous Bacterial Peritonitis and Hepatorenal Syndrome. Hepatology.

[56] Yim H.J., Kim T.H., Suh S.J., Yim S.Y., Jung Y.K., Seo Y.S., Kang S.H., Kim M.Y., Baik S.K., Kim H.S. (2023). Response-Guided Therapy with Cefotaxime, Ceftriaxone, or Ciprofloxacin for Spontaneous Bacterial Peritonitis: A Randomized Trial: A Validation Study of 2021 AASLD Practice Guidance for SBP. Am. J. Gastroenterol..

[57] Facciorusso A., Papagiouvanni I., Cela M., Buccino V.R., Sacco R. (2019). Comparative efficacy of long-term antibiotic treatments in the primary prophylaxis of spontaneous bacterial peritonitis. Liver Int..

[58] Feuerstadt P., Hong S.J., Brandt L.J. (2020). Chronic Rifaximin Use in Cirrhotic Patients Is Associated with Decreased Rate of *C. difficile Infection*. Dig. Dis. Sci..

[59] Pérez-Cameo C., Oriol I., Lung M., Lladó L., Dopazo C., Nuvials X., Los-Arcos I., Sabé N., Castells L., Len O. (2023). Impact of Prophylactic Norfloxacin in Multidrug Resistant Bacterial Infections in the Early Liver Posttransplant Period. Exp. Clin. Transplant..

[60] Hurley J.C. (2021). Selective digestive decontamination, a seemingly effective regimen with individual benefit or a flawed concept with population harm?. Crit. Care.

[61] Myburgh J., Seppelt I.M., Goodman F., Billot L., Correa M., Davis J.S., Gordon A.C., Hammond N.E., Iredell J., Li Q. (2022). Effect of Selective Decontamination of the Digestive Tract on Hospital Mortality in Critically Ill Patients Receiving Mechanical Ventilation. JAMA.

[62] Garcia-Tsao G. (2019). Prophylactic Antibiotics in Cirrhosis: Are They Promoting or Preventing Infections?. Clin. Liver Dis..

[63] Lutz P., Parcina M., Bekeredjian-Ding I., Nischalke H.D., Nattermann J., Sauerbruch T., Hoerauf A., Strassburg C.P., Spengler U. (2014). Impact of Rifaximin on the Frequency and Characteristics of Spontaneous Bacterial Peritonitis in Patients with Liver Cirrhosis and Ascites. PLoS ONE.

[64] Higuera-de-la-Tijera F., Servín-Caamaño A.I., Salas-Gordillo F., Pérez-Hernández J.L., Abdo-Francis J.M., Camacho-Aguilera J., Alla S.N., Jiménez-Ponce F. (2018). Primary Prophylaxis to Prevent the Development of Hepatic Encephalopathy in Cirrhotic Patients with Acute Variceal Bleeding. Can. J. Gastroenterol. Hepatol..

[65] Coronel-Castillo C.E., Contreras-Carmona J., Frati-Munari A.C., Uribe M., Méndez-Sánchez N. (2020). Eficacia de la rifaximina en los diferentes escenarios clínicos de la encefalopatía hepáticaEfficacy of rifaximin in the different clinical scenarios of hepatic encephalopathy. Rev. Gastroenterol. Mex. (Engl. Ed.).

[66] Ponziani F.R., Zocco M.A., D’Aversa F., Pompili M., Gasbarrini A. (2017). Eubiotic properties of rifaximin: Disruption of the traditional concepts in gut microbiota modulation. World J. Gastroenterol..

[67] Patel V.C., Lee S., McPhail M.J.W., Da Silva K., Guilly S., Zamalloa A., Witherden E., Støy S., Manakkat Vijay G.K., Pons N. (2022). Rifaximin-α reduces gut-derived inflammation and mucin degradation in cirrhosis and encephalopathy: RIFSYS randomised controlled trial. J. Hepatol..

[68] Louvet A., Labreuche J., Dao T., Thévenot T., Oberti F., Bureau C., Paupard T., Nguyen-Khac E., Minello A., Bernard-Chabert B. (2023). Effect of Prophylactic Antibiotics on Mortality in Severe Alcohol-Related Hepatitis: A Randomized Clinical Trial. JAMA.

[69] Marciano S., Gutierrez-Acevedo M.N., Barbero S., Del C., Notar L., Agozino M., Fernandez J.L., Anders M.M., Grigera N., Antinucci F. (2023). Norfloxacin prophylaxis effect on multidrug resistance in patients with cirrhosis and bacterial infections. Eur. J. Clin. Microbiol. Infect. Dis..

[70] B Hadi Y., Khan R.S., Lakhani D.A., Khan A.Y., Jannat R.U., Khan A.A., Naqvi S.F., Obeng G., Kupec J.T., Singal A.K. (2023). Antibiotic Prophylaxis for Upper Gastrointestinal Bleed in Liver Cirrhosis; Less May Be More. Dig. Dis. Sci..

[71] Mücke M.M., Mücke V.T., Graf C., Schwarzkopf K.M., Ferstl P.G., Fernandez J., Zeuzem S., Trebicka J., Lange C.M., Herrmann E. (2020). Efficacy of Norfloxacin Prophylaxis to Prevent Spontaneous Bacterial Peritonitis: A Systematic Review and Meta-Analysis. Clin. Transl. Gastroenterol..

[72] Assem M., Elsabaawy M., Abdelrashed M., Elemam S., Khodeer S., Hamed W., Abdelaziz A., El-Azab G. (2016). Efficacy and safety of alternating norfloxacin and rifaximin as primary prophylaxis for spontaneous bacterial peritonitis in cirrhotic ascites: A prospective randomized open-label comparative multicenter study. Hepatol. Int..

[73] Elfert A., Abo Ali L., Soliman S., Ibrahim S., Abd-Elsalam S. (2016). Randomized-controlled trial of rifaximin versus norfloxacin for secondary prophylaxis of spontaneous bacterial peritonitis. Eur. J. Gastroenterol. Hepatol..

[74] Hanouneh M.A., Hanouneh I.A., Hashash J.G., Law R., Esfeh J.M., Lopez R., Hazratjee N., Smith T., Zein N.N. (2012). The role of rifaximin in the primary prophylaxis of spontaneous bacterial peritonitis in patients with liver cirrhosis. J. Clin. Gastroenterol..

[75] Pande C., Kumar A., Sarin S.K. (2012). Addition of probiotics to norfloxacin does not improve efficacy in the prevention of spontaneous bacterial peritonitis: A double-blind placebo-controlled randomized-controlled trial. Eur. J. Gastroenterol. Hepatol..

[76] Fernández J., Navasa M., Planas R., Montoliu S., Monfort D., Soriano G., Vila C., Pardo A., Quintero E., Vargas V. (2007). Primary prophylaxis of spontaneous bacterial peritonitis delays hepatorenal syndrome and improves survival in cirrhosis. Gastroenterology.

[77] Komolafe O., Roberts D., Freeman S.C., Wilson P., Sutton A.J., Cooper N.J., Pavlov C.S., Milne E.J., Hawkins N., Cowlin M. (2020). Antibiotic prophylaxis to prevent spontaneous bacterial peritonitis in people with liver cirrhosis: A network meta-analysis. Cochrane Database Syst. Rev..

[78] Fernández J., Prado V., Trebicka J., Amoros A., Gustot T., Wiest R., Deulofeu C., Garcia E., Acevedo J., Fuhrmann V. (2019). Multidrug-resistant bacterial infections in patients with decompensated cirrhosis and with acute-on-chronic liver failure in Europe. J. Hepatol..

[79] Fernández J., Acevedo J., Castro M., Garcia O., de Lope C.R., Roca D., Pavesi M., Sola E., Moreira L., Silva A. (2012). Prevalence and risk factors of infections by multiresistant bacteria in cirrhosis: A prospective study. Hepatology.

[80] Kremer W.M., Gairing S.J., Kaps L., Ismail E., Kalampoka V., Hilscher M., Michel M., Siegel E., Schattenberg J.M., Galle P.R. (2022). Characteristics of bacterial infections and prevalence of multidrug-resistant bacteria in hospitalized patients with liver cirrhosis in Germany. Ann. Hepatol..

[81] Fernandez J., Piano S., Bartoletti M., Wey E.Q. (2021). Management of bacterial and fungal infections in cirrhosis: The MDRO challenge. J. Hepatol..

[82] Delavy M., Burdet C., Sertour N., Devente S., Docquier J.D., Grall N., Volant S., Ghozlane A., Duval X., Ghozlane A. (2022). A Clinical Study Provides the First Direct Evidence That Interindividual Variations in Fecal β-Lactamase Activity Affect the Gut Mycobiota Dynamics in Response to β-Lactam Antibiotics. mBio.

[83] Shamsaddini A., Gillevet P.M., Acharya C., Fagan A., Gavis E., Sikaroodi M., McGeorge S., Khoruts A., Albhaisi S., Fuchs M. (2021). Impact of Antibiotic Resistance Genes in Gut Microbiome of Patients with Cirrhosis. Gastroenterology.

